# Addressing Iron and Zinc Micronutrient Malnutrition Through Nutrigenomics in Pearl Millet: Advances and Prospects

**DOI:** 10.3389/fgene.2021.723472

**Published:** 2021-11-18

**Authors:** Rakesh K. Srivastava, C. Tara Satyavathi, Mahesh D. Mahendrakar, Ram B. Singh, Sushil Kumar, Mahalingam Govindaraj, Irfan A. Ghazi

**Affiliations:** ^1^ International Crops Research Institute for the Semi-Arid Tropics (ICRISAT), Patancheru, India; ^2^ All India Coordinated Research Project on Pearl Millet (Indian Council of Agricultural Research), Jodhpur, India; ^3^ Department of Agricultural Biotechnology, Anand Agricultural University (AAU), Anand, India; ^4^ Alliance of Bioversity International and the International Center for Tropical Agriculture (CIAT), Cali, Colombia; ^5^ Department of Plant Sciences, School of Life Sciences, University of Hyderabad, Hyderabad, India

**Keywords:** micronutrient malnutrition, iron and zinc deficiency, Pearl millet [*Pennisetum glaucum* (L.) R. Br.], next-generation sequencing (NGS), forward genetics, reverse genetics

## Abstract

Iron (Fe) and zinc (Zn) micronutrient deficiencies are significant health concerns, particularly among the underprivileged and resource-poor people in the semi-arid tropics globally. Pearl millet is regarded as a climate-smart crop with low water and energy footprints. It thrives well under adverse agro-ecologies such as high temperatures and limited rainfall. Pearl millet is regarded as a nutri-cereal owing to health-promoting traits such as high grain Fe and Zn content, metabolizable energy, high antioxidant and polyphenols, high proportion of slowly digestible starches, dietary fibers, and favorable essential amino acid profile compared to many cereals. Higher genetic variability for grain Fe and Zn content has facilitated considerable progress in mapping and mining QTLs, alleles and genes underlying micronutrient metabolism. This has been made possible by developing efficient genetic and genomic resources in pearl millet over the last decade. These include genetic stocks such as bi-parental RIL mapping populations, association mapping panels, chromosome segment substitution lines (CSSLs) and TILLING populations. On the genomics side, considerable progress has been made in generating genomic markers, such as SSR marker repository development. This was followed by the development of a next-generation sequencing-based genome-wide SNP repository. The circa 1,000 genomes re-sequencing project played a significant role. A high-quality reference genome was made available by re-sequencing of world diversity panel, mapping population parents and hybrid parental lines. This mini-review attempts to provide information on the current developments on mapping Fe and Zn content in pearl millet and future outlook.

## Introduction

One-sixth of the global population suffers from hunger which is an unacceptable burden for humankind. A report on “The State of Food Insecurity in the World 2014” created by various organizations suggested that approximately 800 million persons were affected by undernourishment during 2012–2014, a sizable fraction of which are dwelling in the emergent nations (Bohra et al., 2016). A large fraction of the world’s population is consuming rice and wheat as a principal staple. These cereals are relatively not rich in mineral micronutrients which are essential for the human body to maintain basic metabolic rates (White and Broadley, 2009). Worldwide, South Asia and the Indian sub-continent contain 38.9% of the world’s malnourished children, impacting significantly on these economies. The order of decreasing global Fe/Zn malnutrition is as follow; India (38%), Nigeria (7%), Pakistan (6%), China (5%), Indonesia (5%), Bangladesh (4%), Ethiopia (3%), the Democratic Republic of the Congo (3%), Philippines (2%), United Republic of Tanzania (2%), Egypt (2%), Kenya (1%), Uganda (1%) and Sudan (1%) (De Onis et al., 2012).

### Pearl Millet as a Nutrient-Dense Cereal

Pearl millet is a nutrient-rich staple cereal grain with a high amount of carbohydrates, proteins, fats, polyphenols, dietary fibers, vitamins, and micronutrients. Pearl millet grains are a cost-efficient energy source (360 Kcal/100 g) for humans to meet their dietary requirements than sorghum, wheat, rice and maize. Pearl millet has high levels of slowly available starch, high fiber (1.2 g/100 g), and 8–15 times greater α-amylase activity. Pearl millet flour is gluten-free with a low glycemic index and significantly rich in resistant starch, hence the ideal candidate grain for use in the functional-food market worldwide (Ragaee et al., 2006; Saleh et al., 2013). The amino acid profiling indicated that pearl millet grain is rich in histidine, isoleucine, leucine, lysine, methionine, phenylalanine, threonine, tryptophan, and valine that are relatively higher than the other staple cereals. Pearl millet is rich in unsaturated fatty acids with a higher content of nutritionally important omega-3 fatty acids than other cereal grains.

### Grain Fe/Zn Profile of Pearl Millet Relative to Other Cereals

Pearl millet grains are highly nutritious having inorganic nutrients including Fe (22–154 ppm) and Zn (19–121 ppm) (Mahendrakar et al., 2019) with relatively higher amounts of the Fe and Zn than other staple cereal such as wheat (10 ppm), rice (28 ppm), and maize (22 ppm). Genetic biofortification is a way of enhancing nutrition in staple crops in the modern period, and improved food processing and markets will be the backbone of future nutritional security (Govindaraj et al., 2019). Hence breeding for micronutrient-rich pearl millet varieties and their utilization would fulfill the suggested recommended dietary allowance (RDA) of Fe and Zn and also would encourage producing biofortified pearl millet lines (Munyaradzi, 2013). According to a recent study, having 250 g of biofortified pearl millet per day can supply 84% of the RDA for Fe and 100% of the RDA for Zn (Satyavathi et al., 2021).

### Biofortification Options for Alleviating Fe/Zn Malnutrition

Crop biofortification is a viable and economical approach to handle micronutrient malnutrition, specifically in developing countries (Stein et al., 2007; Bouis et al., 2011). Biofortification is a method to improve the micronutrient content of farm harvest. This strategy includes targeting and altering the movement pathways of minerals, i.e., “pulling” nutrients from the earth and “pushing” them into edible plant parts in their bioavailable forms. This pulling and pushing approach can alleviate the malnutrition issues which impact human potential. Compared to currently available tactics namely fortification, supplementation, or dietary diversification, biofortification is a viable means of making available nutrition in remote and rural areas for delivery to the impoverished community having inadequate access to balanced and nutrient-rich food (Bouis et al., 2011).

### Challenges in Biofortification and Phenotyping for grain Fe and Zn

Some of the major challenges in pearl millet biofortification include lack of access to high throughput phenotyping platforms and higher phenotyping costs. Dust contamination in the grain samples, improper/uneven seed set and soil fertility variation introduces error in measurements. The Fe/Zn trait is not simply inherited, it is largely governed by additive gene action, and shows genotype-by-environment interaction especially for small effect alleles. These pose challenges in breeding Fe-Zn dense cultivars. Also, there is a weak negative correlation between grain yield and grain Fe and Zn content, which might become significant in narrow/correlated breeding populations. Further, bioavailability-related issues such as for low phytates in the grains and absence of specific vitamins such as vitamin C in the diet need to be addressed for Fe.

Two highly precise tools namely Atomic Absorption Spectrometry (AAS) and Inductively Coupled Plasma Optical Emission Spectrometry (ICP-OES) are widely used to analyze grain minerals. A detailed methodology of Fe and Zn analysis in pearl millet grain using AAS and ICP-OES is provided by Kumar et al. (2016) and Pujar et al. (2020), respectively. Both AAS and ICP-OES based methodologies are destructive and require acid digested sample preparation which is expensive, time-demanding and low throughput. Therefore, there is a demand for an alternative device for the rapid analysis of massive grain samples. The Energy-dispersive X-ray fluorescence (ED-XRF), an improved version of the XRF method, is a non-destructive technology for speedy analysis of iron (Fe) and zinc (Zn) in grains. In pearl millet, this system has been developed and validated by Govindaraj et al. (2016). This system can handle circa 250–300 samples per day at the cost of US $ < 1.0 per sample compared to the ICP-OES where the analysis cost is about US $18 per sample. The results of earlier studies show that correlations between the ICP-OES and ED-XRF values are significantly higher for both Fe and Zn content (Paltridge et al., 2012; Govindaraj et al., 2016). This can considerably reduce the cost and time for the analysis of Fe and Zn density.

## Development of Genetic and Genomic Resources

### Genetic Resources and Historic Breeding Efforts for Fe and Zn Rich Grains

Large variability has been reported for grain Fe and Zn in germplasm, breeding lines and cultivars. The gene bank of ICRISAT is a promising source of Fe and Zn rich genotypes. A total of 41 and 42 genotypes were richer in Fe (>80 ppm) and Zn (>60 ppm), respectively. While 33 genotypes were found to be a rich source of both minerals (Yadav et al., 2017). The analysis of 297 Iniadi germplasm from Western Africa (Togo, Eastern Ghana, Southern Burkina Faso and Western Benin) has shown wide variability for grain Fe and Zn content (Rai et al., 2008). Several studies at ICRISAT showed a wide range of variability for grain Fe and Zn densities in diverse breeding materials such as Iniadi germplasm accessions (51–121 ppm Fe; 46–87 ppm Zn), population progenies (18.0–135.0 ppm Fe; 22.0–92.0 Zn), inbred parents (30.3–102.0 ppm Fe; 27.4–84.0 ppm Zn), hybrids derived from diverse inbreds (25.8–80.0 ppm Fe; 22.0–70 ppm Zn) and commercial hybrids (31.0–61.0 ppm Fe, 32.0–54.0 ppm Zn) (Govindaraj et al., 2019). Analysis of 281 advanced breeding lines exhibited substantial variability for Fe (35–116 ppm) and Zn (21–80 ppm) (Pujar et al., 2020). ICTP 8203, an open-pollinated variety (OPV) released in India in 1988 had been found to have the highest level of Fe and Zn density (Rai et al., 2013). The efforts in biofortification have yielded nutrient-rich and high-yield cultivars in India (Rai et al., 2014). A world-first high-Fe pearl millet variety “Dhanashakti” (71 ppm Fe) was developed by utilizing the intra-population variability within ICTP 8203. Hybrid ICMH 1201 is also rich in Fe (75 ppm) and under cultivation by the name of Shakti 1201 in Maharashtra and Rajasthan. In India, the first wave of biofortified hybrids are AHB 1200 Fe (ICMH 1202), HHB 299 (ICMH 1203), and Phule Maha Shakti (ICMH 1301) officially released at the National level in India. These biofortified hybrids contain more than 70 ppm Fe and 35 ppm Zn. In general, all the commercial hybrids had 42 and 32 ppm mean Fe and Zn content, respectively (Govindaraj et al., 2019). A mega pearl millet cultivar HHB 67 Improved 2 developed through genomics-assisted breeding has been recently released and notified for cultivation in India. This hybrid is expected to contribute immensely towards food and nutritional security.

### Genomic Resources

#### Molecular Markers

DNA markers like AFLP (Devos et al., 1995) and SSRs (Senthilvel et al., 2008; Sehgal et al., 2012) were applied to detect genetic polymorphism in pearl millet germplasm. SSR markers were also valuable for anchoring molecular linkage maps (Gonzalo et al., 2005) that were densely saturated with DArT (Supriya et al., 2011). EST-SSRs were markers of choice for linkage and association mapping since they are frequently transferable to other related plant species (Varshney et al., 2005; Singh et al., 2011; Singh et al., 2019; Srivastava et al., 2020a). Thousands of functional SSRs have been developed in pearl millet genetics and breeding (Senthilvel et al., 2008; Rajaram et al., 2013), in addition to SSCP-SNP (Bertin et al., 2005), DArT (Supriya et al., 2011; Kumar et al., 2016), CISP and SNP (Sehgal et al., 2012) markers. These molecular tools have successfully been deployed in marker-assisted backcrossing (MABC) to improve “HHB 67 Improved 2” and GHB 538 Improved hybrids from India. Pearl millet genome sequence information of circa 1,000 lines has resulted in genome-wide SNPs (low/mid/high-density panel) for trait mapping, deployment and heterotic gene pools (Varshney et al., 2017; Srivastava et al., 2020b). All these genomic resources have been made available at various public repository platforms. Some key sources include https://cegsb.icrisat.org/ipmgsc/genome.html; http://gigadb.org/dataset/100192; https://www.ncbi.nlm.nih.gov/genome/?term=pearl+millet; https://www.ncbi.nlm.nih.gov/assembly/GCA_002174835.1.

#### Genetic Linkage Mapping and Consensus Maps

**FIGURE 1:**
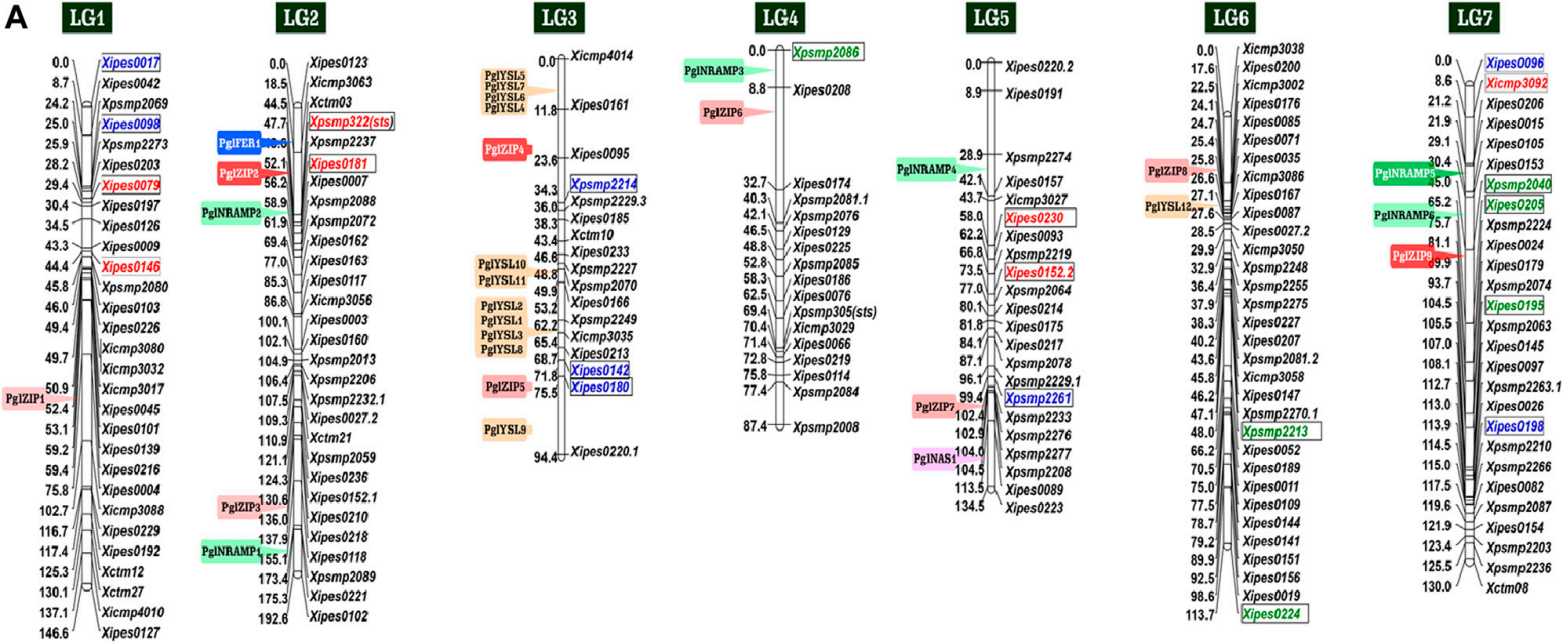
List of the reported linked markers for QTLs, alleles and candidate genes for high grain Fe and Zn content in pearl millet on the consensus map of Rajaram et al. (2013) *Note*: Red font color indicates favorable allele for grain Fe content, green for grain Zn content and blue for both grain Fe and Zn content. The 29 genes for grain Fe and Zn homeostasis belonging to five broad families are in solid color boxes. The five candidate genes for grain Fe and Zn are in solid boxes with white font (Data source: Mahendrakar et al., 2020).

In pearl millet, specific genetic resources were developed to map grain Fe and Zn content. This included forward genetic stocks such as bi-parental mapping populations, association mapping panels, and reverse genetic stocks as the TILLING (Targeting Induced Local Lesions in Genomes) populations. Using bi-parental mapping populations. Kumar et al. (2016) developed a linkage map of 1749 cM that was generated with 305 markers (96 codominant SSRs and 208 dominant DArTs) with inter-marker distance of 5.73 cM based on a previously reported consensus map (Rajaram et al., 2013). Kumar et al. (2018) used another population to construct a genetic linkage map with DArT and SSR markers using 317 RIL progenies derived from the (ICMS 8511-S1-17-2-1-1-B-P03 × AIMP 92901-S1-183-2-2-B-08) cross. Recently, Mahendrakar et al. (2020) reported unique genes on seven linkage groups. Figure 1 summarizes the latest mapping results for GFeC and GZnC in pearl millet from different studies. In addition, reverse genetic stocks as TILLING populations are being developed at ICRISAT in the three genetic backgrounds including that of world reference germplasm Tift 23DB1-P1-P5 for functional genomics related to Fe and Zn and other important traits.

## Marker-Trait Association for Grain Iron and Zinc Density

Significant efforts have been made to develop a fine linkage map and Fe-Zn QTLs in pearl millet (Kumar et al., 2016). Hundreds of markers were employed to generate a linkage map for mapping QTLs for GFeC and GZnC using 106 RILs (F_6_) derived from ICMB 841-P3 × 863B-P2 cross in pearl millet (Kumar et al., 2016). Using phenotypic data from two environments, two co-localized QTLs for Fe and Zn content on LG3 were detected. Similarly, for open-pollinated (OP) seeds, the QTL analysis detected two Fe-QTLs on LG3 and LG5, and two Zn-QTLs on LG3 and LG7. The total phenotypic variance for Fe-QTL and Zn-QTLs in OP seeds was 16 and 42%, respectively (Kumar et al., 2016). In another analogous study, 11 Fe-QTLs and 8 Zn-QTLs have been identified, among them three major QTLs on LG1 and two on LG7 for both Fe and Zn were co-mapped in pearl millet (Kumar et al., 2018). The phenotypic variance explained by different Fe-QTLs and Zn-QTLs ranged between 9.0–31.9% and 9.4–30.4%, respectively (Table 1). A total of 46 QTLs in pearl millet were detected with phenotypic variance ranged from 6.7 to 20.5% and 5.7–26.4% for GFeC and GZnC QTLs, respectively (Kumar et al., 2016; Kumar et al., 2018). Although, linkage analysis in most of the investigations indicates the existence of genes/QTLs at a distance of 5.73–23.0 cM from the flanking markers for MABC programs and functional validation of candidate genes (Vengadessan et al., 2013). In addition to SSR markers, genome-wide SNPs are more suited for genome-wide association studies (GWAS), and genomic selection (GS) and QTL mapping for Fe/Zn traits in pearl millet (Srivastava et al., 2020a). A panel of pearl millet germplasm lines was evaluated under the different agro-climates using SSRs for identification of strong maker trait associations (MTAs) using GWAS, in which *Xicmp3092* marker exhibited a significant association with GFeC on LG7 and *Xpsmp2086*, *Xpsmp2213* and *Xipes0224* markers were showed association with GZnC on LG4 and LG6, respectively (Anuradha et al., 2017). Conserved association for GFeC and GZnC was demonstrated by *Xipes0180*, *Xpsmp2261*, and *Xipes0096* markers on LG3, LG5, and LG7, respectively (Anuradha et al., 2017). Using 144 multi-parent advance generation inter-cross (MAGIC) plus population, Descalsota et al. (2018) identified SNPs for grain Fe and Zn. They identified few Fe and Zn homeostasis genes such as OsMTP6, OsNAS3, OsMT2D, OsVIT1 and OsNRAMP7 which were localized with QTLs. This information may be helpful in the biofortification of Fe and Zn in pearl millet. The LG3 grain Fe and Zn QTL (Kumar et al., 2016) interval was sequenced for the development of SNP markers. A 4-SNP panel is being used in the breeding programs for rejecting lines with poor grain Fe and Zn content.

**TABLE 1:** Summary of various studies for high grain Fe (GFeC) and Zn (GZnC) content in pearl millet.

Sl. no.	Objective	Plant accessions/population used	Range of GFeC (ppm)	Range of GZnC (ppm)	DNA markers used	Number of identified QTLs/genes/MTAs linked with GFeC and GZnC	Associated markers/candidate genes	Phenotypic variance (R^2^)	References
1.	Identification and validation of candidate genes underlying GFeC and GZnC metabolism	Two contrast parents of RILs population AIMP 92,901 (high grain Fe and Zn), and ICMS 8511 (low grain Fe and Zn)	22.93–154.5	19.31–121.00	N/A	5 Candidate gene includes; PglFER1, PglZIP2, PglZIP4, PglNramp5, and PglZIP9 controlling with GFeC and GZnC	Ferritin-like gene, *PglFER1 (Fe)*, *PglZIP and PglNRAMP* (Fe and Zn)	N/A	Mahendrakar et al. (2020)
2.	To map QTLs linked with GFeC and GZnC in an iniadi-derived immortal pearl millet mapping population	A panel of 317 F_6_ recombinant inbred lines (RILs) derived from the (ICMS 8511-S1-17-2-1-1-B-P03 × AIMP 92901-S1-183-2-2-B-08) cross	20–131	18–110	196 (177 DArTs & 19 SSRs)	Total 11 QTLs detected for GFeC and 8 for GZnC. 3 QTLs co-mapped for both GFeC-GZnC observed, 1 on LG1 and 2 on LG7	Fe-Zn: pgpb10531-pgpb9130, Fe: pgpb8427-pgpb13221 pgpb11938-pgpb8987 Zn: Xipes198-pgpb8427 pgpb12329-pgpb9721	GFeC: 9.0–31.9% (cumulative 74%). GZnC: 9.4–30.4% (cumulative 65%)	Kumar et al. (2018)
3.	Genome-wide association mapping of QTLs for Fe & Zn	An association mapping panel comprised of 130 diverse lines (B-, R- & advanced breeding lines)	32.3–111.9	26.6–73.7	267 markers (250 SSRs & 17 genic markers)	SSRs associated with both grain GZnC and GZnC on LG3, LG5 and LG7	Xipes 0810 (aspartic proteinase gene) Xpsmp 2261 (intergenic region) Xipes 0096	11.4, 13.3 and 11.3% R^2^, respectively	Anuradha et al. (2017)
4.	Mapping of QTLs controlling high GFeC and GZnC in self and open-pollinated grains of pearl millet	A set of 144 RILs derived from the (ICMB 841-P3 × 863B-P2) cross	a) Fe-Self 28.4–124	a) Zn-Self 28.7–119.8	305 (96 SSRs & 208 DArTs)	In selfed seed, a 2 co-localized QTL for GFeC and GZnC on LG3. b) In open pollinated seeds 2 QTLs for GFeC on LG 3 & LG5, and 2 QTLs for GZnC on LG 3 & LG7.	Fe & Zn: Xpsmp2214-Xipes0142. OP Fe: Xpsmp2214-Xipes0142 Pgbp5908-Pgpb6674 OP Zn: Xpsmp2214-Xipes0142. Xpsmp2040-Pgpb10727	Fe: 19.0%; Zn: 36.0% Fe: 16.0%; Zn: 42.0%	Kumar et al. (2016)
			b) Fe-OP 22.4–77.4	b) Zn- OP 21.9–73.7					

N/A = not applicable.

### Evolution of Genotyping Platforms

With the advent and recent advancement of next-generation sequencing (NGS) technology with affordable cost, high-throughput genotyping platforms (HTGP) have emerged. These HTGPs revolutionized genotyping assays in a cost-efficient manner and rapid protocols. Using NGS-based genotyping platforms, assaying for genomic variations, high-resolution linkage mapping, identification of candidate gene(s) and natural allelic variants underlying for QTLs controlling yield-related traits have become regular practices for crop species. In pearl millet, various next-generation sequencing-based genotyping platforms have been developed and optimized. These can help generate thousands of SNPs in various genetic stocks, breeding populations, and landraces. Genotyping-by-sequencing (GbS) is a speedy, economical and reduced representation sequencing method that is frequently deployed for genome-wide genetic variability profiling in cereal crops (Elshire et al., 2011). Over singe (*Ape*K1) enzyme GbS, two enzyme combinations (SphI and *Mlu*C1) double digest restriction associated DNA (ddRAD) was found to be superior (Srivastava et al., 2016) and was used to fine map the LG2 drought tolerance QTL (Srivastava et al., 2017). The other genotyping platforms that are available include tunable-GbS (tGbS) (Ott et al., 2017; Liang et al., 2018), DArT-seq (Allan et al., 2020); and RNase H2 enzyme-dependent amplicon sequencing (rhAmpSeq) (Dobosy et al., 2011).

### Transcriptome Assemblies and Candidate Genes

**FIGURE 2:**
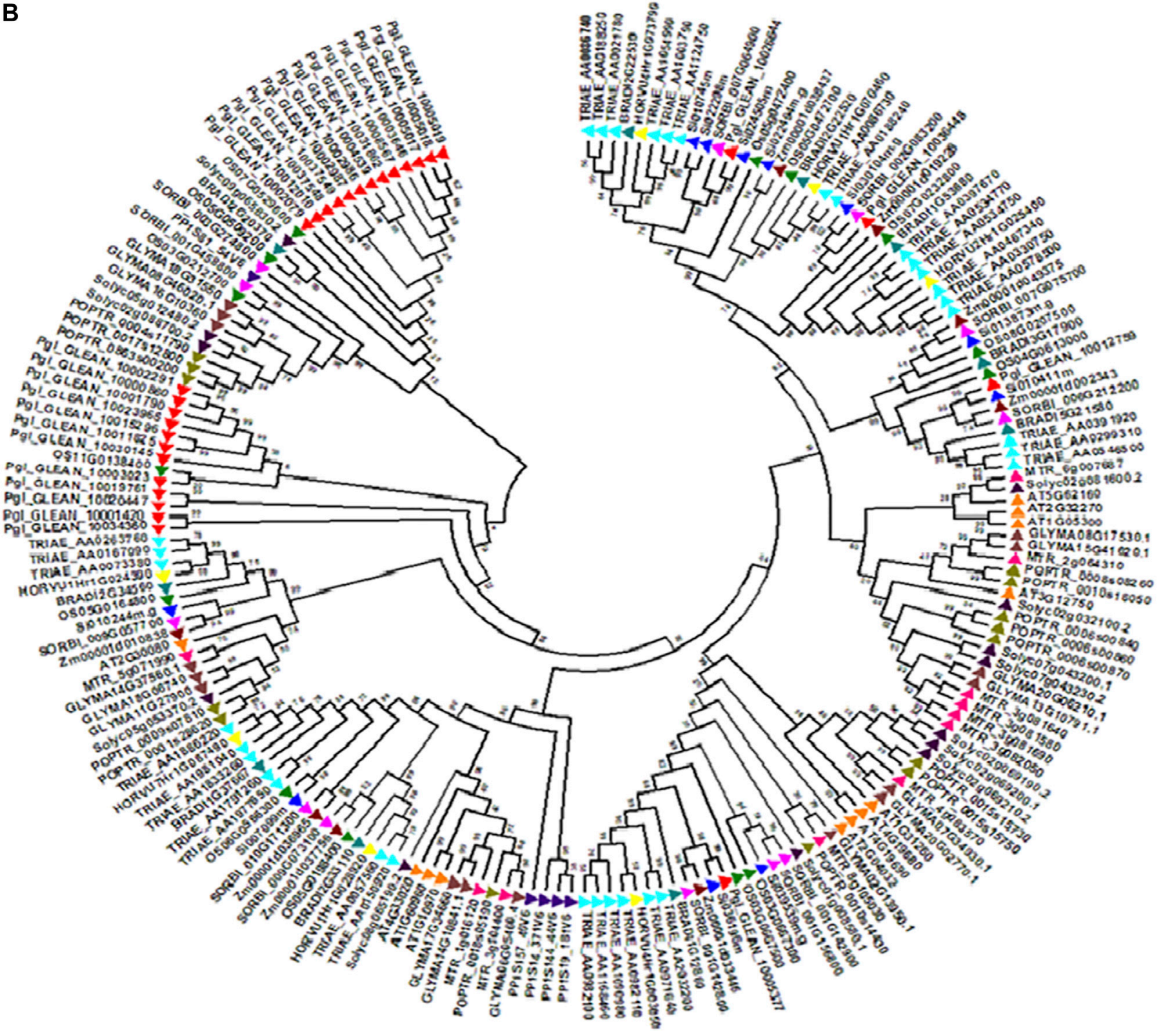
Phylogeny tree of reported 29 pearl millet candidate genes (prefix Pgl) for Fe and Zn metabolism with that of other cereal crops showing evolutionary relationship and ortholog events with other cereal crops (Data source: Mahendrakar et al., 2020).

**FIGURE 3:**
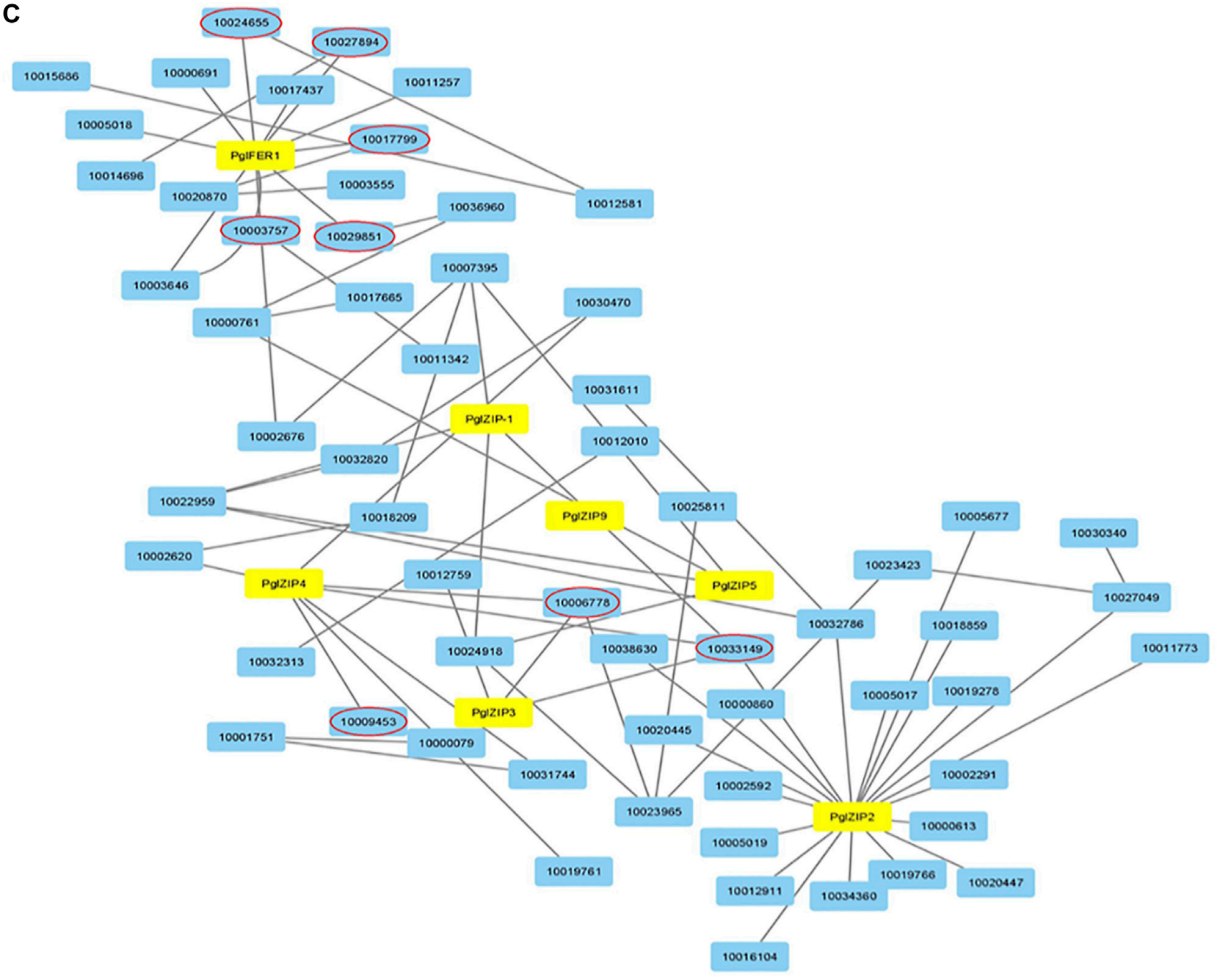
Protein-protein interaction (PPI) network for 7 putative proteins. Yellow colored nodes have been short listed from 29 unique proteins (Mahendrakar et al., 2020) and other interacted nodes (red circled) involved in Fe and Zn metabolism and transport and other functions like ATP binding, phosphotransferase activity, carbohydrate metabolism and hexokinase activity (Data source: Mahendrakar et al., 2020).

To discover underlying mineral homeostasis, transcriptomics may pave the way to gene discovery and increased biosynthesis of useful nutritional products (Hirai et al., 2004). Knowledge of the genes controlling specific stages in the Fe and Zn metabolic pathways is relatively meagre in pearl millet. Recently, using transcriptomic studies, the key candidate genes were discovered for grain Fe and Zn metabolism in pearl millet (Mahendrakar et al., 2020). A total of 29 unique genes were used for spatio-temporal characterization using expression studies for different developmental stages in contrasting genotypes for GFeC and GZnC. Tissue and stage-specific expressions of Fe and Zn genes in contrasting genotypes for GFeC and GZnC were recorded (Mahendrakar et al., 2020). Results revealed that PglZIP, PglNRAMP, PglYSL, and PglFER family genes were candidates for GFeC and GZnC. The evolutionary relationship of the 29 unique genes showed ortholog events with other related cereals studied (Figure 2). To further support the reported candidate genes, protein-protein interaction (PPI) was studied. The PglFER and PglZIP gene families interacted with various other proteins associated with Fe and Zn metabolism along with metabolism of carbohydrates, hexokinase, protein kinase, ATP binding protein and phosphotransferase activity (Figure 3). Ferritin-like gene (PglFER-1) was found to be the most potent candidate gene for GFeC. For further validation, genomic regions underlying GFeC and GZnC were deciphered by annotating QTL regions for grain Fe and Zn densities. Expressed genes were correlated with major QTL co-localized on LG7 for GFeC and GZnC (Mahendrakar et al., 2020). The study provided a useful understanding of different Fe and Zn metabolism gene homologs and laid a foundation for functional dissection. Ogo et al. (2011) reported overexpression of OsIRO2 resulted in enhanced accumulation of Fe in brown rice grain even in calcareous soil and further recorded high Zn in grains. By overexpressing the soybean-ferritin gene, a high iron rice variety called IR68144 was developed in which iron concentration was increased by 3.7 fold in polished rice grain (Vasconcelos et al., 2003). Another group overexpressed soybean ferritin gene (*soyfer*1) in the same rice variety and interbred with Swarna rice variety resulting in a new variety with 2.54 fold and 1.54 fold more Fe and Zn content in rice grain, respectively (Paul et al., 2014).

## Conclusion and Future Outlook

To enhance the genetic gains for grain micronutrient contents and yield, it is essential to understand the germplasm, breeding lines, and functional and translational genomics. The availability of cost-efficient NGS-based technologies have revolutionized whole-genome sequencing and transcriptome analysis. This has led to genome-wide genetic dissections of untapped valuable alleles. The identified genomic regions/QTLs controlling micronutrient accumulation and homeostasis into elite hybrid parental lines and OPVs may pave the way to breed high grain Fe/Zn cultivars using low density markers such as the available 4-SNP panel and pan-genome marker-assisted selection schemes. The promising lines with favourable alleles bred by using such technologies may be useful for generating new cultivars which accumulate all major alleles regulating grain iron and zinc metabolism. The Indian Council of Agricultural Research (ICAR) minimum standards for Fe (42 ppm) and Zn (32 ppm) in pearl millet cultivar release policy and the product profiles support use of molecular markers in mainstreaming these traits. National and international gene banks should sequentially genotype the germplasm to map all essential nutritional traits to explore traits specific germplasm and its novel genes. Thus, with the help of nutrigenomic advances, the next generation of nutrient-dense farmer preferred cultivars may be bred with precision and efficiency in pearl millet.
